# Toward Patient Specific Models of Pediatric IVDs: A Parametric Study of IVD Mechanical Properties

**DOI:** 10.3389/fbioe.2021.632408

**Published:** 2021-02-15

**Authors:** Edmund Pickering, Peter Pivonka, J. Paige Little

**Affiliations:** ^1^School of Mechanical, Medical and Process Engineering, Queensland University of Technology, Brisbane, QLD, Australia; ^2^Biomechanics and Spine Research Group, Centre for Children's Health Research, Queensland University of Technology, Brisbane, QLD, Australia

**Keywords:** intervertebral disc, pediatric, finite element, patient specific, stiffness

## Abstract

Patient specific finite element (FE) modeling of the pediatric spine is an important challenge which offers to revolutionize the treatment of pediatric spinal pathologies, for example adolescent idiopathic scoliosis (AIS). In particular, modeling of the intervertebral disc (IVD) is a unique challenge due to its structural and mechanical complexity. This is compounded by limited ability to non-invasively interrogate key mechanical parameters of a patient's IVD. In this work, we seek to better understand the link between mechanical properties and mechanical behavior of patient specific FE models of the pediatric lumbar spine. A parametric study of IVD parameter was conducted, coupled with insights from current knowledge of the pediatric IVD. In particular, the combined effects of parameters was investigated. Recommendations are made toward areas of importance in patient specific FE modeling of the pediatric IVD. In particular, collagen fiber bundles of the IVD are found to dominate IVD mechanical behavior and are thus recommended as an area of primary focus for patient specific FE models. In addition, areas requiring further experimental research are identified. This work provides a valuable building block toward the development of patient specific models of the pediatric spine.

## 1. Introduction

Development of patient specific spine models is of increased interest in the treatment of pediatric spinal pathologies. Such models aim to revolutionize clinical practice by providing practitioners with detailed predictions of a patient's spinal biomechanics. For example, such models are already showing promise in predicting outcomes of corrective interventions for AIS (Little and Adam, 2011; Vergari et al., 2015; Aubin et al., 2018).

The accuracy of these models is underpinned by the fidelity in which the patient specific geometry and patient specific material parameters are represented. Here, patient specific geometry can be readily extracted from medical scans (Strickland et al., 2011; Finley et al., 2018) (including the use of pre-operative scans, Little and Adam, 2015). In contrast, patient specific material parameters cannot be directly interrogated and are thus a greater challenge. As a substitute, these are commonly extracted from experimental biomechanics studies. However, a majority of research on IVD biomechanics focuses on adult cases, thus there is a paucity of material data for pediatric IVDs. As such, most FE models of the pediatric IVD use material parameters extracted from studies on adult IVDs (for example Sairyo et al., 2006; Little et al., 2008; Cahill et al., 2012; Dong et al., 2013).

It thus becomes important to understand how the material parameters and related mechanics of the pediatric IVD differs from those of adults. We address this challenge by first understanding how pediatric IVD material parameters differ from adults, followed by exploring how this affects the overall IVD mechanics. Focus will be place on adolescents (i.e., minimum age of 10 year). Further, focus will be placed on the annulus fibrosus (AF) as this is the main source of variance in FE IVD models (it being well-accepted that in FE models of the young, healthy IVD, the nucleus pulposus (NP) can be treated as an incompressible fluid Fagan et al., 2002a; Rohlmann et al., 2006; Little et al., 2008; Dong et al., 2013). To achieve this, a detailed literature review on the biomechanics of the AF and those parameters which affect mechanical behavior was conducted. This was complemented by a parametric study which explored how AF parameters affected the stiffness of the IVD under axial rotation, flexion, extension and lateral bending. This cumulated in overall recommendations for FE modeling of pediatric IVDs.

## 2. The Pediatric IVD

While, most IVD research focuses on the case of healthy adults and/or degenerative IVDs, this work places a special focus on pediatric IVDs—an overlooked area. It thus becomes important to build an understanding of the biomechanics of the IVD, focusing on those parameters relevant for mechanical behavior. There however is a paucity of studies on those parameters relevant for modeling of pediatric IVDs. Below, we aim to highlight key findings, insights and hypothesis of the pediatric IVD biomechanics relevant for FE modeling. Focus will be placed on the AF fiber angle, AF fiber stiffness and AF ground matrix stiffness, as these are the key variables in such FE models. In addition, while the NP is not of focus in this study, development changes will be briefly addressed. Focus will not be placed on the geometry of the IVD as in patient specific models, these are derived from medical imaging. Finally, focus is not placed on the aged or degenerate IVD as these deviate from the healthy case and are outside of the scope of this research (Urban et al., 2000; Sharabi et al., 2018).

### 2.1. Embryology and IVD Development

The AF and NP have different embryonic origins; the AF deriving from the sclerotome while the NP derives from the notochord (Sivakamasundari and Lufkin, 2012). By full term however, the fetal IVD exhibits the structure of an adult IVD (Walmsley, 1953). Thus, it is reasonable to conclude that the modeling approach used in adult IVDs (Shirazi-Adl et al., 1986) is valid for pediatric IVDs.

### 2.2. Stiffness of Collagen Fiber Bundles

Collagen fiber bundles of the IVD are responsible for carrying tensile loads, thus the fiber stiffness becomes a key parameter. Fiber stiffness can be considered either by studying the elastic properties of individual fiber bundles (Holzapfel et al., 2005; Zhu et al., 2008), or by study the stiffness of larger IVD sections (i.e., multiple fiber bundles embedded in ground matrix) and extracting the fiber stiffness (Galante, 1967; Wu and Yao, 1976; Little et al., 2010).

During growth from a pediatric to adult IVD, several changes can be expected within individual collagen fiber bundles. The bundle thickness will increase (Marchand and Ahmed, 1990; Langlais et al., 2019), the number of individual bundles will increase (Marchand and Ahmed, 1990), and chemical changes can be expected (Galante, 1967; Buckwalter, 1995; Sharabi et al., 2018). These all link to the mechanical behavior of the fiber bundles, but provide limited information on the overall stiffness of the collagen fiber bundles themselves. Further, no study has explored the age-related stiffness of a single fiber bundle. Thus, it becomes relevant to consider the embodied stiffness of a larger section of the IVD.

The most detailed study on fiber stiffness and age was conducted by Galante (1967). In this, AF sections were tested to a set tensile load. No significant trends were observed for samples over 26 years of age, matching other findings (Ebara et al., 1996; Holzapfel et al., 2005). However, for ages under 26 years, sample elongation increased steadily. At 10 years of age, samples exhibited 50% greater elongation, in comparison to those over 26 years. This is equivalent to a 33% reduction in stiffness. Two key factors should be noted here. First, the AF consists of collagen fibers embedded in a ground matrix. The results presented are thus the combined stiffness of the collagen fibers and the ground matrix. However, under tension the fibers are significantly stiffer than the ground matrix, thus the results here can be assumed directly applicable to the fibers themselves. Second, the stiffness here is a combined effect of the fiber elastic modulus, fiber spacing and fiber cross-sectional area. This is in-fact beneficial as consideration need not be given to these individual parameters, rather the fiber stiffness can be considered on a whole. Thus, in summary, for IVDs over 26 year, fiber stiffness remains constant, however, for ages below 26 years, the fiber stiffness decreases gradually. For samples of 10 years age, the fiber stiffness can be 33% less than that of IVDs over 26 years.

### 2.3. Fiber Angle

In the adult lumbar IVD, fiber angle varies with both location and radial depth (Cassidy et al., 1989; Holzapfel et al., 2005), however it is generally accepted that the mean fiber angle is 30° (Holzapfel et al., 2005; Michalek, 2019). Holzapfel et al. (2005) reported a 95% prediction interval of approximately ±15° for fiber angle.

Considering average fiber angles during development, in a study of fetal IVDs, Hickey and Hukins (1980) observed no trend in fiber angles between a conception ages of 10–25 weeks. Further, the fiber angles observed by Hickey and Hukins aligned to studies of adult IVDs (Cassidy et al., 1989; Holzapfel et al., 2005). Thus, it is reasonable to argue that average the fiber angle does not change appreciably during development.

Adding weight to this argument, Michalek (2019) proposed a growth-based model to predict fiber angles. In this, Michalek assumed that growth of the AF initiates from a thin cylinder with constant angles and thus hypothesized that the fiber angles can be fully predicted based upon IVD geometry. The resulting model showed good alignment to experimental results. Crucially, it has been shown that during growth, the IVD height and diameter increase at the same rate (Taylor, 1975). For this case, the Michalek model would predict the average fiber angles in the pediatric IVD to match that of an adult. A further advantage of the Michalek model is that it may predict patient-specific fiber orientation in IVDs with atypical geometries; for example in AIS patients which have increased disc height compared to the population average (Ponrartana et al., 2016).

Combining the facts that (1) fiber angle observations of fetuses (Hickey and Hukins, 1980) align with those of adults (Cassidy et al., 1989; Holzapfel et al., 2005), and (2) the IVD exhibits approximate linear scaling during growth (Taylor, 1975) for which the Michalek model (Michalek, 2019) would predict similar fiber angle, we conclude fiber angle distributions in the pediatric IVD would align to those of the adult IVD. Thus, the fiber angle in pediatric patients can be expected to be independent of age, with an mean angle of 30°, and with natural population variances inline with those observed by Holzapfel et al. (2005).

### 2.4. Ground Matrix

To our best knowledge, no study has explored age-dependent changes of ground matrix mechanical properties. This is a limitation in patient specific models of pediatric IVDs, which will require additional experimental work to address. An alternative approach would be to focus on changes in the biochemistry and composition of the ground matrix, and make inferences about the age-dependent mechanical properties. For example, the pediatric IVD has a lower concentration of glycoproteins (Galante, 1967). We however recommend against this approach. Mechanical behavior is function of both composition and structure. Thus, making assumptions on mechanical behavior based upon composition is an uncertain process.

### 2.5. Nucleus Pulposus

The NP is composed predominately of water (70–90%), proteoglycans (65% of dry weight), collagen (20% of dry weight), elastic fibers and other proteins (Bogduk, 2005). Due to its high water content and low resistance to shear (Iatridis et al., 1996, 1997), FE models of the NP generally considered it to behave like a hydrostatic fluid (Fagan et al., 2002a; Rohlmann et al., 2006; Little et al., 2008; Dong et al., 2013). During aging, the water content of the IVD degreases while the collagen content increases (Urban and McMullin, 1988; Urban et al., 2000). In turn, the pressure within the NP reduces (Urban and McMullin, 1988). Further, the transition from AF to NP becomes unclear (Urban et al., 2000). However, these changes occur in the aging IVD, while a lack of literature is present on how the pediatric NP differ from the healthy adult case. As the healthy adult NP approximates a hydrostatic fluid, it is reasonable to assume the young NP behaves in a similar nature. Although, the variance in hydrostatic pressure of the young NP remains an open question.

### 2.6. Summary

Based upon the above investigation, the following conclusions can be drawn. First, from an early age (i.e., full term fetus) the IVD exhibits the structure of an adult IVD (Walmsley, 1953), suggesting that modeling approaches used for adult IVDs will be valid for pediatric IVDs. Overall fiber stiffness increases with age in an approximately linear fashion up to 26 years. Stiffness of collagen fibers in a 10 year old IVD can be expected to be 33% less than that of a healthy adult (Galante, 1967). There is however little data on how fiber stiffness varies within particular age brackets, thus this remains an open question. Observations of fiber angles in fetal IVDs match those of adults (Hickey and Hukins, 1980; Cassidy et al., 1989; Holzapfel et al., 2005). This suggests little difference across age group and thus standard population variance can be assumed (Holzapfel et al., 2005). With respect to the ground matrix, no data was found on its stiffness in young age groups, thus this remains an open question. While, much detail is available on the biochemical changes (Galante, 1967; Sharabi et al., 2018), we advise caution in inferring mechanical properties from these as mechanical behavior is both a function of composition and structure. Finally, while numerous structural and composition changes occur in the NP during aging, it is reasonable to assume the pediatric IVD behaves in a similar nature to the young, healthy, adult IVD.

## 3. Method

### 3.1. Geometry

An FE model representative of the L1-2 IVD was generated from the computed 'tomography (CT) dataset of the Visible Man (The Visible Human Project, US National Library of Medicine). The IVD geometry was identified from CT by manually extracting keypoints of the superior and inferior surface using an in-house MATLAB code. These keypoints were then imported into an in-house Python code, which defined the IVD geometry based upon a parametric description of endplate geometry (Little et al., 2007). Further detail on this process is described in Little et al. (2007) and Little and Adam (2012).

As this study is focused on mechanical properties, we elected to use geometry extracted from the Visible Man, as this is a well-studied geometry (Cooper et al., 2001; Little et al., 2008; Lavecchia et al., 2018). Use of a commonly studied and publicly available geometry will increase translatability of findings. Further, in patient specific modeling, any influence of a patient's specific geometry can be extracted from medical images. As the adult and pediatric IVD show similar structure, we argue that any findings can be translated to the pediatric domain (incorporating patient specific geometry).

### 3.2. Finite Element Model

FE modeling of the IVD is a balance between model fidelity (i.e., how closely the model represents the true biological case) and model complexity. Introduction of higher fidelity representations increases the model complexity. In this work, we employ well-established methods of representing the AF ground matrix, AF collagen fibers and NP. Through this we seek to balance the needs of fidelity and complexity.

**Figure 1:**
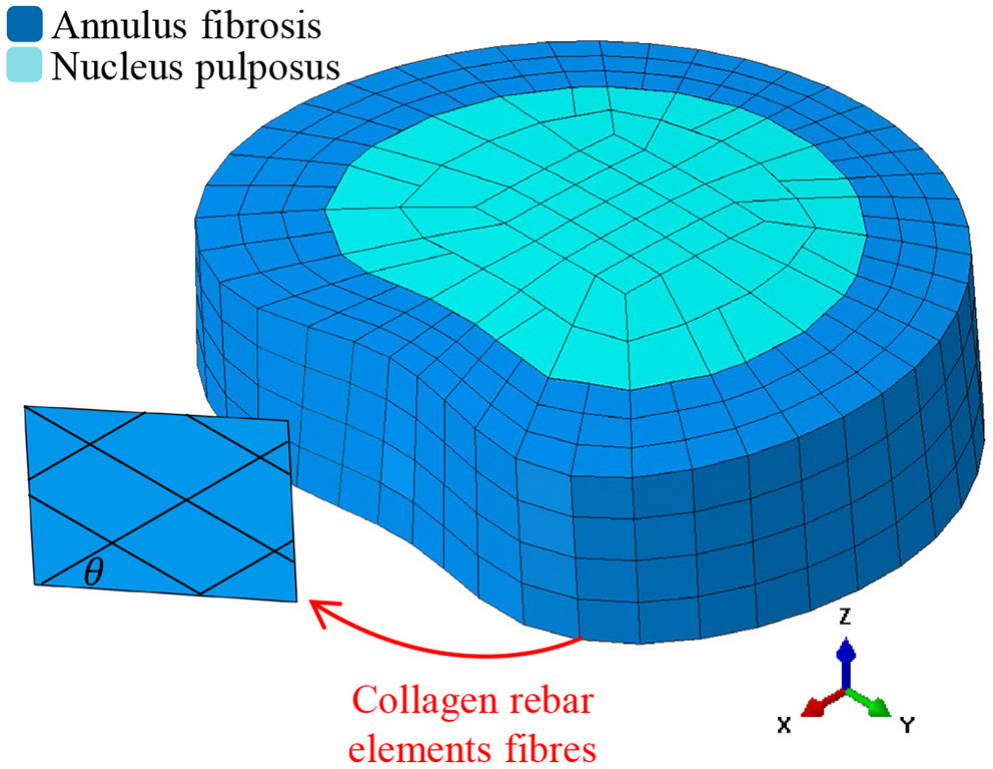
Schematic representation of the IVD finite element model.

In this work, we use a previously validated IVD model (Little et al., 2008). As our process for modeling the IVD has been previously described in detail (Little et al., 2008), we will only provide a brief description here. A schematic representation of the FE mesh is shown in Figure 1, which follows the meshing approach of most common IVD FE models (Shirazi-Adl et al., 1986; Smit et al., 1997; Little et al., 2008; Dreischarf et al., 2014). The AF was modeled as a ground matrix with reinforcing collagen fibers (Shirazi-Adl et al., 1986; Little et al., 2008; Dong et al., 2013). The AF ground matrix was modeled by three concentric rings of three dimensional (3D) solid continuum elements (see Figure 1). The collagen fibers were represented as tension-only rebar elements located on the hoop faces of the AF at an angle of θ (where θ represents the fiber angle from the horizontal plane as shown in Figure 1). Each hoop-face consisted of two-layers of alternate angled rebar elements, equivalent to eight lamellae (Shirazi-Adl et al., 1986; Little et al., 2008; Dong et al., 2013). The collagen fibers were assigned an elastic modulus of *E* with a cross-section selected such that the volume of the collagen fibers was 25% that of the AF (Marchand and Ahmed, 1990) The NP was represented using 3D hydrostatic elements (Fagan et al., 2002a; Rohlmann et al., 2006; Little et al., 2008; Dong et al., 2013) with a pressure of 0.25 MPa prior to loading (Wilke et al., 1999; Meir et al., 2007). Details of the NP, AF ground matrix and collagen fibers are given in Table 1 including element type, material model and properties.

**Table 1:** Elements, constitutive models, and properties of the IVD.

	**Element type**	**Material model**	**Material properties**
NP	3D, 4-node fluid element	Hydrostatic fluid	Incompressible (Nachemson, 1960; Goel et al., 1995)
AF ground matrix	3D, 8-node, solid element	Hyperelastic Mooney-Rivlin	*C*_10_ = 0.7 (Natali and Meroi, 1990; Little et al., 2008)
			*C*_01_ = 0.2 (Natali and Meroi, 1990; Little et al., 2008)
Collagen fibers	Rebar tension-only	Linear elastic, tension only	*E* = 500 MPa^*^ (Ueno and Liu, 1987)
			Volume fraction, *V*_*f*_ = 0.25 (Marchand and Ahmed, 1990)
			θ = 30° (Shirazi-Adl et al., 1986)

* *The stiffness of the collagen fibers is proportional to E·V_f_. As such, the stiffness of the fibers will be controlled by varying E*.

Replicating realistic loading on a model of a single the IVD is challenging as the deformation of the IVD is governed by the mechanics of the spinal column. Further, spinal motion is driven by the motion and mechanics of the facet joints and the intervertebral joint at each spinal motion segment. In simulating the IVD in isolation, it is important for the boundary and loading conditions to create motion in the IVD which mimics that observed in the full spine. Many studies have described the deformation of the IVD using the instentaneous axis of rotation (IAR). In this, the motion of the superior endplate is tracked relative to the inferior endplate and at each instant the center of rotation is found. *In vivo* (Pearcy and Bogduk, 1988), *ex vivo* (Cossette et al., 1971), and *in silico* (Schmidt et al., 2008) studies have all explored the IAR in IVDs of the lumbar spine.

In this work, we take guidance from Schmidt et al. (2008) who explored the IAR of a lumbar functional spinal unit via FE modeling. Schmidt et al. found that for common motions (i.e., axial rotation, flexion, extension, and lateral bending) under low moments, the IAR locus was near the centroid of the IVD. For larger moments (up to 7.5 N), the IAR shifted, in a manner largely governed by spinous process interactions. Adapting this, it was assumed that the IAR was located at the centroid of the IVD. To replicate this, nodes of the inferior endplate were fixed, while nodes of the superior endplate were pinned via rigid beam elements to a nodes located at the centroid of the IVD. This node was then pin supported, causing the IVD to deform about the IAR. Four motions were investigated in this study; axial rotation, flexion, extension and lateral bending. In each case, these motions were produced through the application of a 7.5 Nm moment to the superior endplate, over 30 substeps. Loading magnitudes were selected in-line with other studies (Dreischarf et al., 2014; Newell et al., 2017; Finley et al., 2018).

### 3.3. Study Design

The objective of this work is to understand the combined influence of IVD parameters on predicted rotational stiffness. To achieve this, the study was split into three studies. In the first study the influence of individual properties on the IVD was explored, through this the properties which had a significant contribution to the IVD stiffness were identified. In the second study, the combined influence of these significant properties was investigated. In the final study, the independence/convolution of combined parameters was explored. As previously discussed, the parameters which are studied are those relating to the AF as these dictate the stiffness of the IVD. The parameters which are explored are fiber angle, fiber stiffness, ground matrix *C*_10_ and ground matrix *C*_01_.

#### 3.3.1. Study One: What Are the Individual Influences of IVD Parameters?

In the first study, parameters of the IVD were varied individually. A large range of IVD parameters are found in literature (Galante, 1967; Cassidy et al., 1989; Marchand and Ahmed, 1990; Ebara et al., 1996; Gu et al., 1999; Holzapfel et al., 2005; Zhu et al., 2008), thus to capture most cases, each parameters was varied over a range of ±50% (with nine cases across the range). For each case, the four loading scenarios were modeled, and the stiffness extracted, by fitting a linear trend to the moment-rotation curve. Each stiffness was normalized against the stiffness of the baseline IVD (i.e., the IVD with parameters presented in Table 1). The parameters which had a greater than 10% influence over the IVD stiffness were deemed significant.

#### 3.3.2. Study Two: What Are the Combined Influences of IVD Parameters?

In the second study, the combined effect of IVD parameters was investigated. In this, parameters were varied together across a full range of their potential combinations. For example, if two parameters were deemed significant, then this would result in a total of 9^2^ = 81 simulations, likewise if four parameters were deemed significant, this would require 9^4^ = 6561 simulations. This is why the first stage sought to identify those parameters which were significant, thus reducing the total number of simulations required.

#### 3.3.3. Study Three: Is the Combined Influence Independent or Convoluted?

Study three explored if the effect of changing one parameter is independent of changes in other parameters. By way of explanation, assume in the first parametric study that a change in parameter *A* results in a stiffness increase of 50% while a change in parameter *B* results in a stiffness increase of 20%. If the effects of there parameters are independent, then changing both *A* and *B* together would result in an overall stiffness change of 80% (i.e., 1.5 × 1.2 = 1.8). If the influence of parameters was convoluted (i.e., not independent), then the combined influence would be other than 80%. Understanding independence of parameters on the overall IVD stiffness is important because, if independent, the full effect of a parameter can be understood from the parametric study presented in study one. However, if parameters are convoluted, then the effect of one parameters is dependent on the other parameters and thus a broader awareness must be maintained when specifying parameters in a patient specific FE model.

In a more generalized form, let *P*_θ_, *P*_*k*_, *P*_*C*_10__ be the percentage change in IVD stiffness caused by a change in fiber angle, fiber stiffness, and ground matrix C_10_, respectively (i.e., results from study 1). Likewise, let *P*_θ,*k*,*C*_10__ be the percentage change in IVD stiffness from combined changes of fiber angle, fiber stiffness and ground matrix C_10_ (i.e., results from study 2). If these parameters are independent then:

(1) $$\begin{array}{l} P_{\theta ,k,C_{10}}=\left(1+P_{\theta }\right)\left(1+P_{k}\right)\left(1+P_{C_{10}}\right)-1 \end{array}$$

In study three, the independence of parameters was tested by using Equation (1) to determine the expected combined effects of parameters from study 1, assuming independence. These were then compared to the effects predicted in study 2, and the coefficient of determination (R^2^) was calculated.

## 4. Results

### 4.1. Rotation Behavior of the Baseline IVD

**Figure 2:**
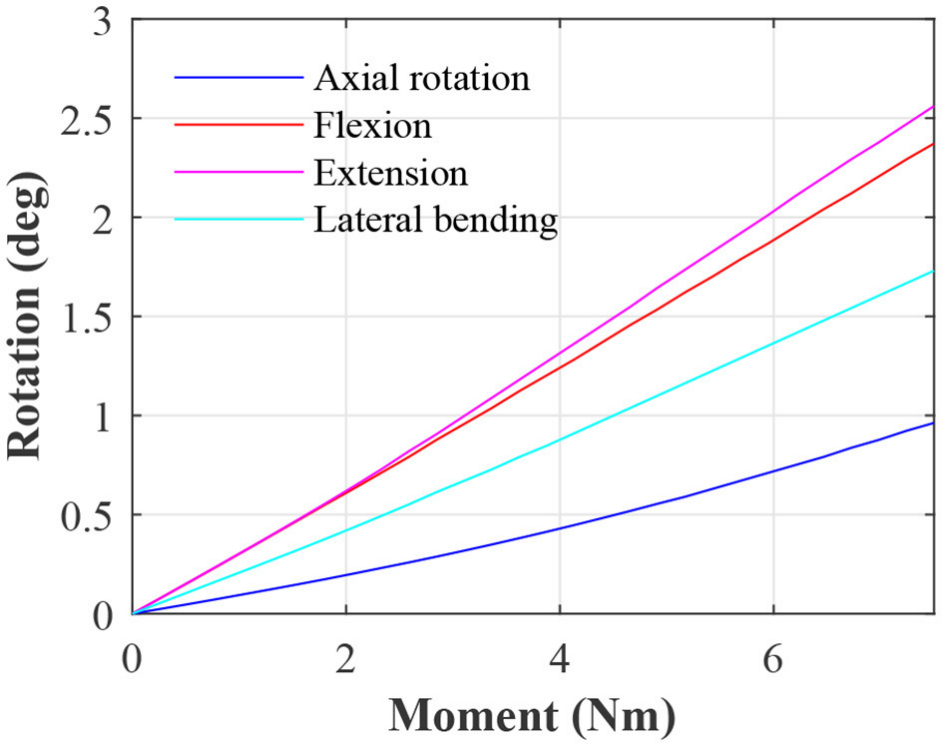
Moment-rotation data for the IVD with default material properties.

To allow benchmarking against other studies, moment-rotations data for an IVD with the baseline parameters is presented in Figure 2.

### 4.2. Study One: What Are the Individual Influences of IVD Parameters?

**Figure 3:**
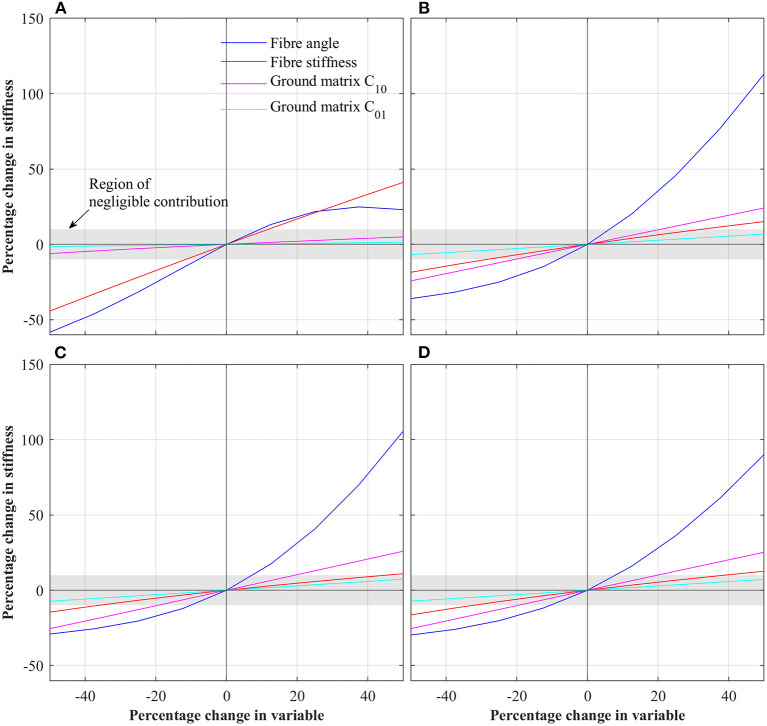
Percentage change in IVD stiffness as a function of changing individual parameters for the different loading conditions. **(A)** Axial rotation. **(B)** Flexion. **(C)** Extension. **(D)** Lateral bending.

Figure 3 shows the results of the first parametric study in which each parameter was varied by ±50%. For each case, stiffness was extracted by linear regression. To normalize stiffness values, the percentage change in stiffness compared to the baseline IVD is reported. To determine those parameters which have a significant effect on IVD stiffness, a threshold of ±10% was set (i.e., those parameters which when varied by ±50% caused a change in IVD stiffness of greater than ±10% where deemed significant). For the case of axial rotation, fiber angle and fiber stiffness were deemed significant. For the cases of flexion, extension and lateral bending, fiber angle, fiber stiffness and ground matrix C_10_ were deemed significant.

### 4.3. Study Two: What Are the Combined Influences of IVD Parameters?

**Figure 4:**
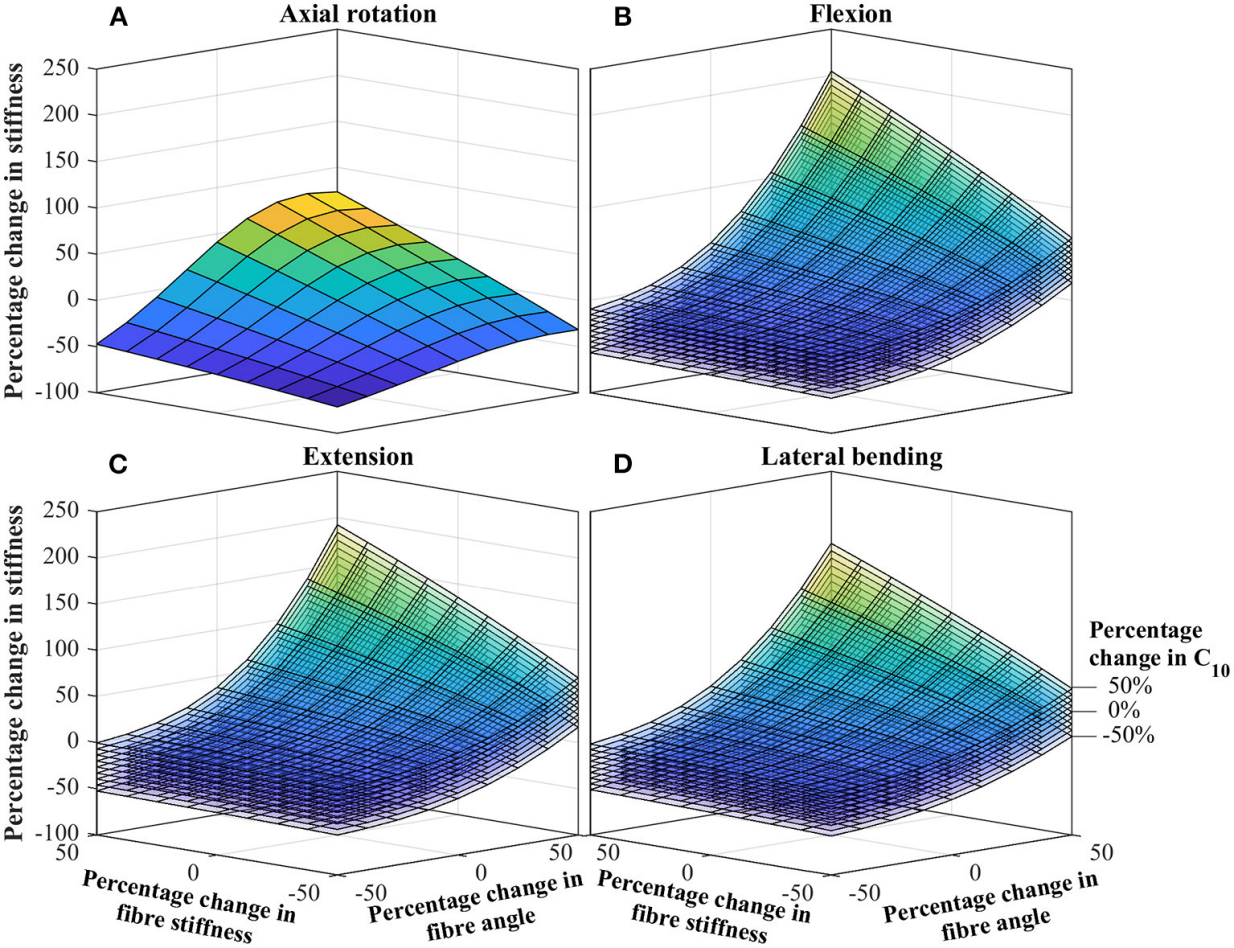
Percentage change in IVD stiffness as a function of combined changing of significant parameters. **(A)** Axial rotation. **(B)** Flexion. **(C)** Extension. **(D)** Lateral bending. The stack of curves in **(B–D)** are for the percentage change in *C*_10_ as identified in **(D)**. Contour plots of the same data is presented in the Supplementary Material.

Parameters deemed significant from study one, were progressed to study two, in which the combined influence of varying parameters were explored. Figure 4 shows curves for the percentage change in stiffness, as a function of the significant parameter changes. For the axial rotation case (Figure 4A), this is represented by a single curve, while for the other cases (Figures 4B,C) this is represented by a stack of curves. The results presented in Figure 4 are semi-quantitative, for full quantitative data, contour plots of the same data is presented in the Supplementary Material.

### 4.4. Study Three: Is the Combined Influence Independent or Convoluted?

**Figure 5:**
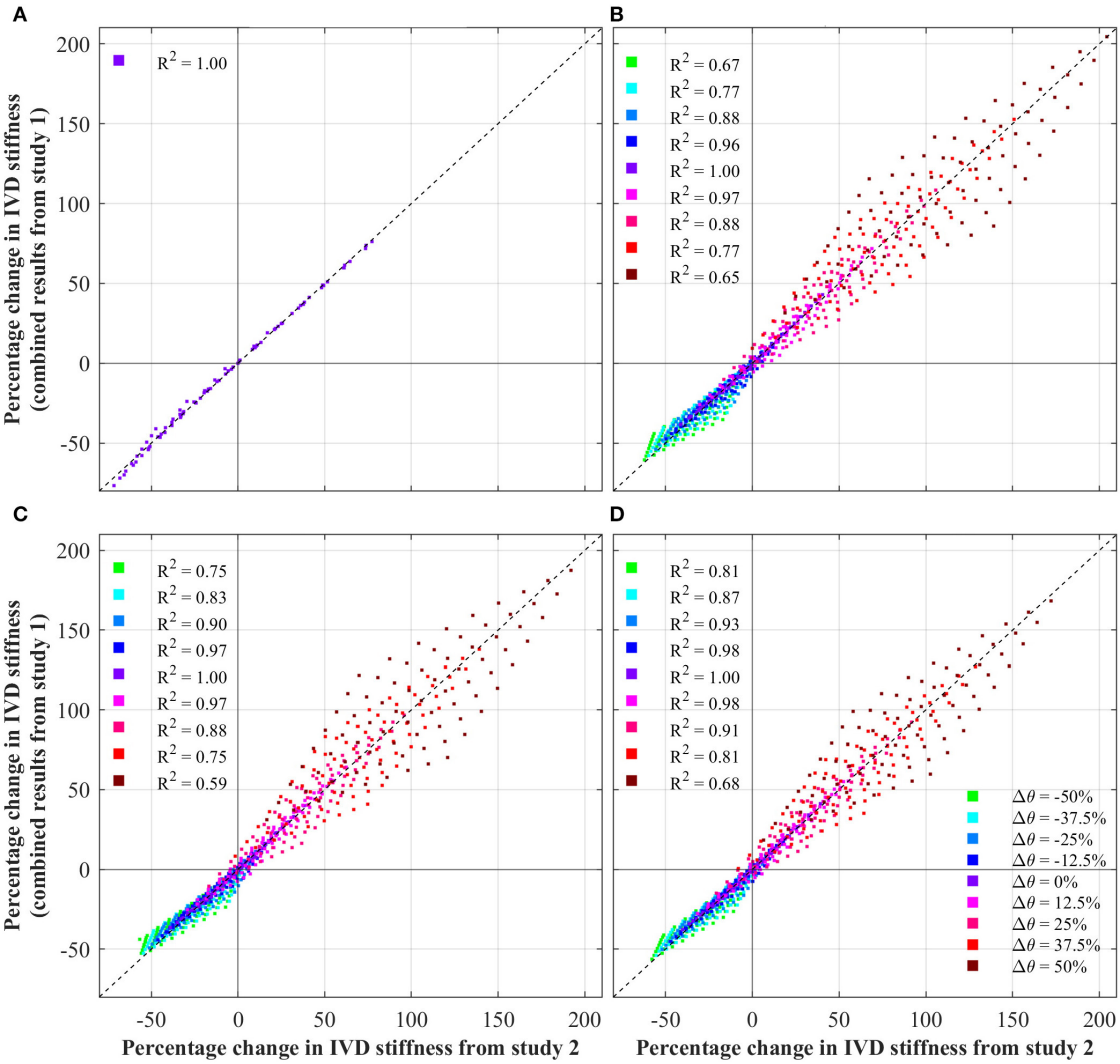
Test of independence of IVD parameters, showing the change in IVD stiffness from the combined study (study 2) against that predicted by Equation (1). Note, in the case of axial rotation, ground matrix C_10_ was not a significant variable, and thus was not modified. For ease of interpretation, each data points is colored based upon its Δθ. Additionally, the coefficient of determination (R^2^) is shown for each θ, against the fit indicated by the dashed black line. **(A)** Axial rotation. **(B)** Flexion. **(C)** Extension. **(D)** Lateral bending.

Figure 5 presents the results from study 3, which explored the independence of parameters effect of IVD stiffness. Each data point represents a single simulation from study 2, plotting the change in IVD stiffness measured in study two, against the predicted change in stiffness (assuming independence, using Equation 1). Independence is indicated by the locus of data points sitting on the dashed line. For readability, data points are labeled based upon the θ parameters, as this was observed to have the biggest influence on independence.

## 5. Discussion

In this work, we used a previously validated FE model of an IVD to investigate the influence of biomechanical parameters on the overall stiffness of the IVD. Specifically, our study focused on parameters of the AF as these are the main source of variance in IVD models [it being well-accepted that for FE modeling of the young, healthy IVD, the NP can be modeled as an incompressible gel, Fagan et al., 2002a; Rohlmann et al., 2006; Little et al., 2008; Dong et al., 2013]. In the below discussion we explore how these parameters influence the overall stiffness of the IVD, in particular we identify which parameters are of greater significance, and we explore how the influence of these parameters combine. The focus here is to build a deeper understanding of how IVD parameters effect mechanical stiffness, with a focus toward patient specific models of the pediatric spine.

### 5.1. Rotation Behavior of Baseline IVD

The moment-rotation curves from the baseline IVD (see Figure 2) are in line with other studies in both terms of shape and magnitude. From numerical models, it is generally well-accepted that the IVD has a roughly linear moment-rotation response (Fagan et al., 2002b); this should not be confused with a functional spinal unit, which presents large non-linear behavior (Ayturk et al., 2010; Dreischarf et al., 2014). The magnitude of the moment-rotation response is in line with other studies (Dreischarf et al., 2014; Mills and Sarigul-Klijn, 2019). However, some numerical studies present IVDs with a noticeably lower stiffness (Fagan et al., 2002b), but this can be attributed to softer material properties. For example Fagan et al. (2002b) used an elastic modulus of 4 MPa for the AF ground matrix, which is significantly softer than the Mooney-Rivlin coefficients used in this study, which are approximately equivalent to an elastic modulus of 5.2 MPa for small deformation. For confirmation, the baseline IVD was simulated with the same AF ground matrix properties as in Fagan et al. which resulted in a similar stiffness.

### 5.2. What Are the Individual Influences of IVD Parameters?

Overwhelmingly, for all loading scenarios, fiber parameters dominate IVD stiffness (see Figure 3). These align with other studies on the influence of fiber properties within the IVD (Fagan et al., 2002b; Guerin and Elliott, 2007). Under axial rotation, the effect of fiber angle and fiber stiffness are much greater than that of the ground matrix C_10_ and C_01_. Across the total variable range, the fiber angle and fiber stiffness had an absolute maximum effect of 44.25 and 58.33%, respectively. Conversely, the maximum absolute effect of C_10_ and C_01_ were 6.1 and 1.5%, respectively. Thus, it is concluded that during axial rotation, ground matrix stiffness parameters are of little importance.

The behavior under flexion, extension and lateral bending were similar, as these directions are all rotations about axes tangential to the transverse plane. As such, for simplicity, discussion here will focus on flexion, but the overall conclusions are transferable to extension and lateral bending. Under flexion, the fiber angle has the greatest impact, especially for positive increases in fiber angle, where a 50% in fiber angle resulted in a 113% increase in IVD stiffness. Conversely, for a −50% change in fiber angle, the IVD stiffness is reduce by 36%. Interestingly, the second most significant parameter is ground matrix C_10_, with a maximum absolute effect of 24.2%, followed by the fiber stiffness with a maximum absolute effect of 18.5%. From these results, it is apparent that the most significant parameters affecting the IVD stiffness is the fiber angle (for flexion, extension, and lateral bending). Thus, in patient specific modeling, high-fidelity representation of the collagen fibers is imperative. Likewise, in efforts to calibrate a model to match experimental findings, fiber angle should be the first target.

For all loading conditions, the effect of the AF ground matrix *C*_01_ parameter was of negligible significance. In the first parametric study, the maximum effect of *C*_01_ was 1.5% for axial rotation, 6.8% for flexion, 7.3% for extension, and 7.2% for lateral bending. By way of explanation, the larger *C*_10_ term of the Mooney-Rivlin model dominates the lesser *C*_01_ term, such that changes in *C*_01_ have little impact on the overall stiffness of the ground matrix, and thus the overall IVD. Both experimental and modeling studies have shown the strains experienced in the AF are in the range of milli-strains (Shirazi-Adl et al., 1986; Disney et al., 2019; Tavana et al., 2020), whereas hyper-elastic models are relevant for larger strains. For example, considering the Mooney-Rivlin parameters used in this study, under uniaxial loading, the ground matrix behaves roughly linear for strains below 50%. Based upon this, we propose that during patient specific modeling (especially those focused on pseudo-static loading) the applicability of higher-order constitutive models of the AF ground matrix be considered.

### 5.3. What Are the Combined Influences of IVD Parameters?

Data from the combined influence study, Figure 4, demonstrates how the effect of individual parameters combine to give a much greater overall effect. Most noteworthy, a clear relationship is observed between the influence of fiber angle and fiber stiffness. For low fiber angles, the fiber stiffness appears to have little effect; conversely for large fiber angles, the fiber stiffness effect is dramatically magnified (and vice versa). The relationship is most apparent for flexion, extension and bending. For all cases, the influence of C_10_ appears uniform, having between a ±25 and ±35% on the overall stiffness, with little dependence of other parameters. This strengthens the earlier statements regarding the importance of high-fidelity fiber modeling, as a strong relation between the fiber angle and fiber stiffness is observed.

### 5.4. Study Three: Is the Combined Influence Independent or Convoluted?

As discussed above, an apparent relationship is observed between the effect of fiber angle and fiber stiffness. This is explored in Figure 5 which tests the independence of IVD parameters by plotting the changes in IVD stiffness from the combined parametric study (study 2) against the change in IVD stiffness predicted by Equation (1) (assuming independence). Under axial rotation it can be seen that changes in fiber angle and fiber stiffness have an independent effect on IVD stiffness. This is shown by data points sitting on the dashed line, and is further demonstrated by an R^2^ of 1.00. This is important because it indicates that variances in one parameters don't impact the effect of the other parameter. Another way of understanding this, the relative effect of errors in one parameters will no magnify errors in another parameter.

However, under flexion, extension and later bending, a degree of dependence is observed between the various parameters, indicated by the locus of data points not sitting on the dashed line. This shows that there is a degree of convolution between the effect of different parameters on IVD stiffness. Interestingly, the degree of convolution is governed by the fiber angle. For a Δθ = 0%, the impact of different parameters follows Equation (1) (indicated by an R^2^ of 1.00), however as the fiber angle increases or decreases, the convolution increases. This is particularly apparent for large fiber angles, where the convolution is greatest (indicated by an R^2^ of 0.68). This reinforces the discussion from section 5.3, in which it was observed that increases in fiber angle, magnify the effect of changes in fiber stiffness.

Clearly, there is a convolution between fiber angle and fiber stiffness, which causes a magnified effect for flexion, extension and bending (especially for increases in fiber angle). This is relevant to patient specific modeling of the IVD as this demonstrates the independence/convolution of IVD parameters under different loadings. In the case of axial rotation, parameters of fiber angle and fiber stiffness are independent. This means that a complete understanding of the effect of these parameters, can be achieved by studying the results of the first parametric study (Figure 3). However, in the case of flexion, extension and bending, the effect of parameters are convoluted, which means that when considering the effects of various parameters, one must consider the convoluted behavior.

### 5.5. Inferences for Pediatric Patient Specific Modeling

Based upon these findings, we propose the following. (1) For patient specific models of the IVD, primary focus should be placed on accurate representation of fiber parameters. Specifically, for models focused on axial rotation both fiber angle and stiffness should be given equal attention, for other motions fiber angle be given greater attention. (2) Non-invasive interrogation of fiber angle for individual patients is a current challenge, thus new techniques need to be developed to incorporate these patient specific fiber parameters. Ultrasonography has been demonstrated as one technique for interrogating parameters such as lamellar number and thickness (both which affect fiber stiffness) (Langlais et al., 2019), this could be one avenue for further investigation. Further models such as the Michalek (2019) growth model could offer an avenue for integrating patient specific fiber angles. (3) The ground matrix parameters are of little significance for axial rotation, but are of secondary interest in for other motions. Currently, limited experimental data is available for ground matrix properties of pediatric IVDs, making this an area requiring further investigation.

For modeling of the pediatric IVD, our earlier literature search (section 2) concluded that fiber angles in the pediatric IVD likely match those of the adult. Thus, improved fidelity of fiber angle in patient specific models will improve the overall quality of predictions. Here, the (Michalek, 2019) growth model could offer potential and should be explored.

With respect to fiber stiffness, Galante (1967) demonstrated that patients of <26 years of age had reduced stiffness, up to a 33% reduction in fiber stiffness for patients of 10 years of age. This will be most significant during axial rotation, where a 33% reduction in fiber stiffness, would result in an approximate 28% reduction in IVD stiffness. For flexion, extension and lateral bending, this reduction would be −12, −9.1, and −10%, respectively. We propose that Galante (1967) can be used as a guide for patient specific fiber properties in pediatric models.

Considerations such as these are of increased significance when dealing with specific pediatric pathologies. Generally, little information is available on IVD parameters in such patients. The discussion above acts as further guidance to required areas of focus in such modeling. For example, in AIS, quantitative variation in the size and orientation of collagen fiber bundles has been observed in opposite sides of the AF (Roberts et al., 1993). Such changes would be expected to cause large variance in overall IVD behavior. Naturally, further investigation into IVD parameters in pediatric pathologies will aid in FE modeling of these pathologies.

### 5.6. Limitations

In any FE model of the IVD certain limitations are inherent and should be noted. First, this study used geometry from a single IVD, from which general conclusions were drawn. For this, it is argued that any effects of patient specific geometries can be extracted from medical imaging, thus focusing on a single, well-studied geometry will aid in translatability of results. Second, this study focused on a single constitutive model for all components, balancing model fidelity and complexity. For example, higher fidelity models can be considered which incorporate non-linear collagen fiber properties (Haut and Little, 1972; Sharabi et al., 2018), variable fiber distributions (Malandrino et al., 2013), visco-elastic effects (Castro and Alves, 2020), and osmotic effects (Cegoñino et al., 2014; Castro and Alves, 2020). While, these higher fidelity models would consider additional behaviors, they would also introduce additional parameters and more complexity. As this study is interested in comparing the relative contributions of individual IVD parameters, it becomes pertinent to focus on simpler constitutive models. Further, we argue that irrespective of the constitutive model, the general findings in the parametric study are valid. Next, this study focused on an IVD in isolation. This has the advantage of only incorporate IVD behavior, but neglects the impact of the whole functional spinal unit. To account for this, the IAR method was used to embed realistic motions. Finally, the influence of NP hydrostatic pressure was not investigated in this study, but has been demonstrated the impact the mechanics and stiffness of the IVD, this should be considered in conjunction with the findings presented here.

## 6. Conclusion

This work has investigated the influence of various mechanical parameters on the stiffness behavior of the IVD under various loading conditions. Notably, while other studies have investigated the individual influence of individual parameters (Fagan et al., 2002b), this works has investigated the combined influence of parameters, demonstrating how these effects are convoluted, and can be magnified. These findings were contextualized with respect to the pediatric IVD, resulting in recommendations for patient specific models of the pediatric IVD and areas requiring further research. This work provides a valuable building block toward the development of such patient specific models.

## Data Availability Statement

The original contributions presented in the study are included in the article/Supplementary Material, further inquiries can be directed to the corresponding author/s.

## Author Contributions

JL developed the initial IVD model. EP conducted the parametric study, results analysis and wrote the manuscript. PP and JL reviewed the manuscript and provided valuable inputs. All authors contributed to the article and approved the submitted version.

## Conflict of Interest

The authors declare that the research was conducted in the absence of any commercial or financial relationships that could be construed as a potential conflict of interest.
