# Supplementary material for: Toward Patient Specific Models of Pediatric IVDs: A Parametric Study of IVD Mechanical Properties
Source: Front Bioeng Biotechnol. 2021 Feb 15;9:632408. doi: 10.3389/fbioe.2021.632408 (PMC7917075; doi:10.3389/fbioe.2021.632408)
Supplement: Supplementary file 1 [file Data_Sheet_1.PDF]

Supplementary Material

STUDY TWO: WHAT ARE THE COMBINED INFLUENCES OF IVD PARAMETERS

Figures S1-S4 are contour representations of the data from Figure 4 of the manuscript. These are provided as a quantitative representation of the data.

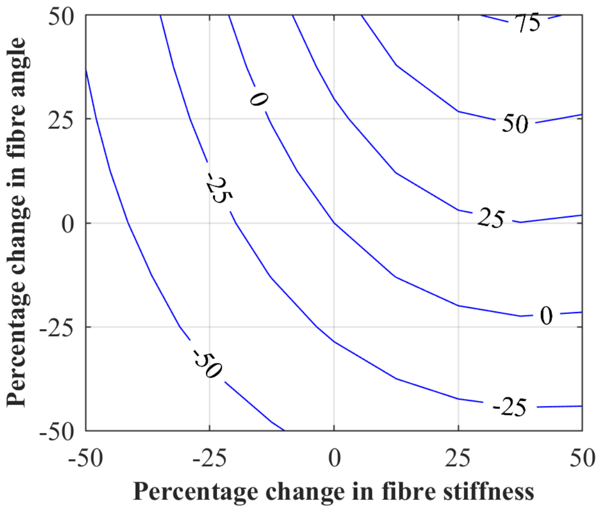

Figure S1. Percentage change in IVD stiffness under axial rotation as a function of changes in the significant parameters.

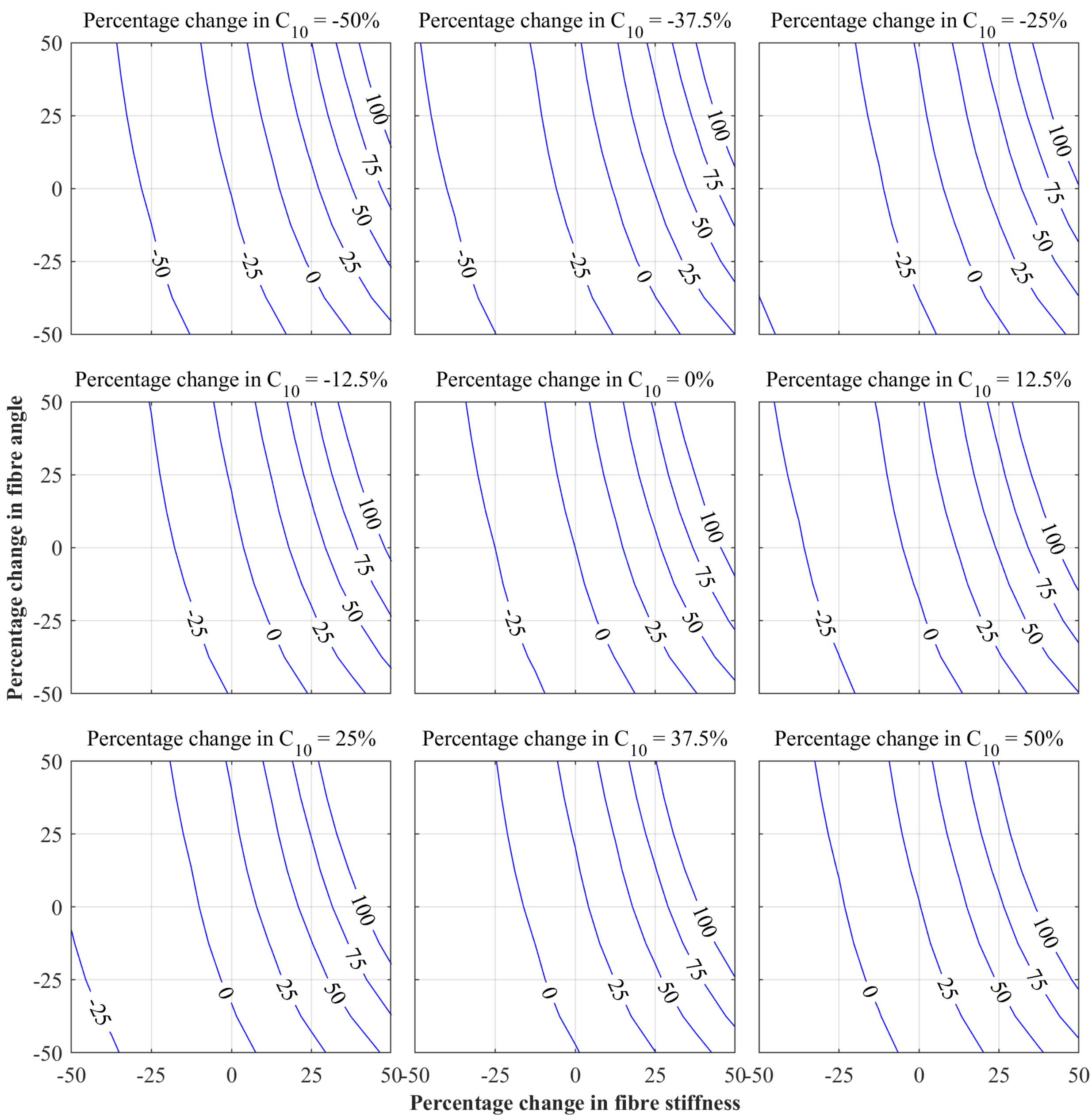

Figure S2. Percentage change in IVD stiffness under flexion as a function of changes in the significant parameters.

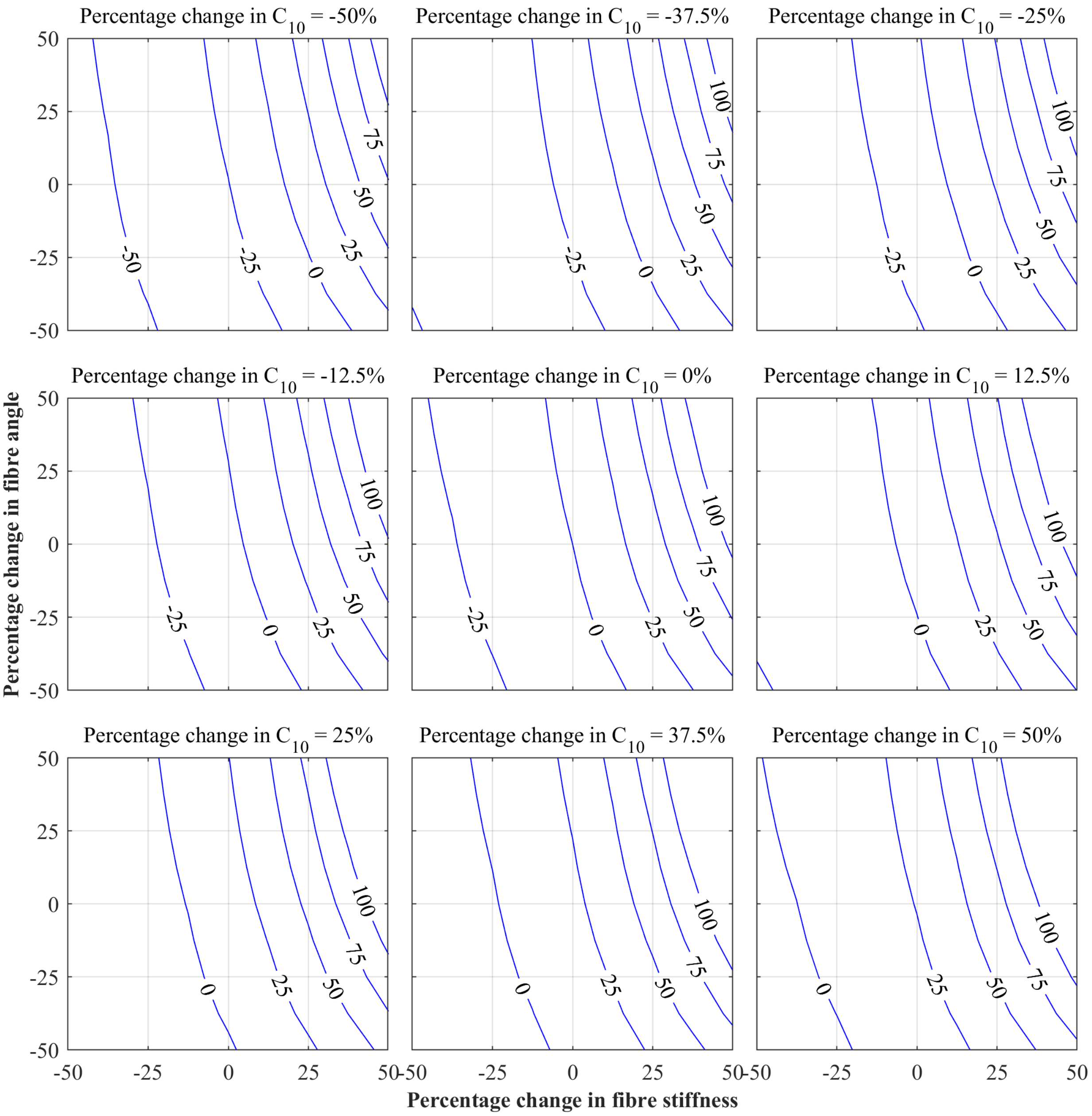

Figure S3. Percentage change in IVD stiffness under extension as a function of changes in the significant parameters.

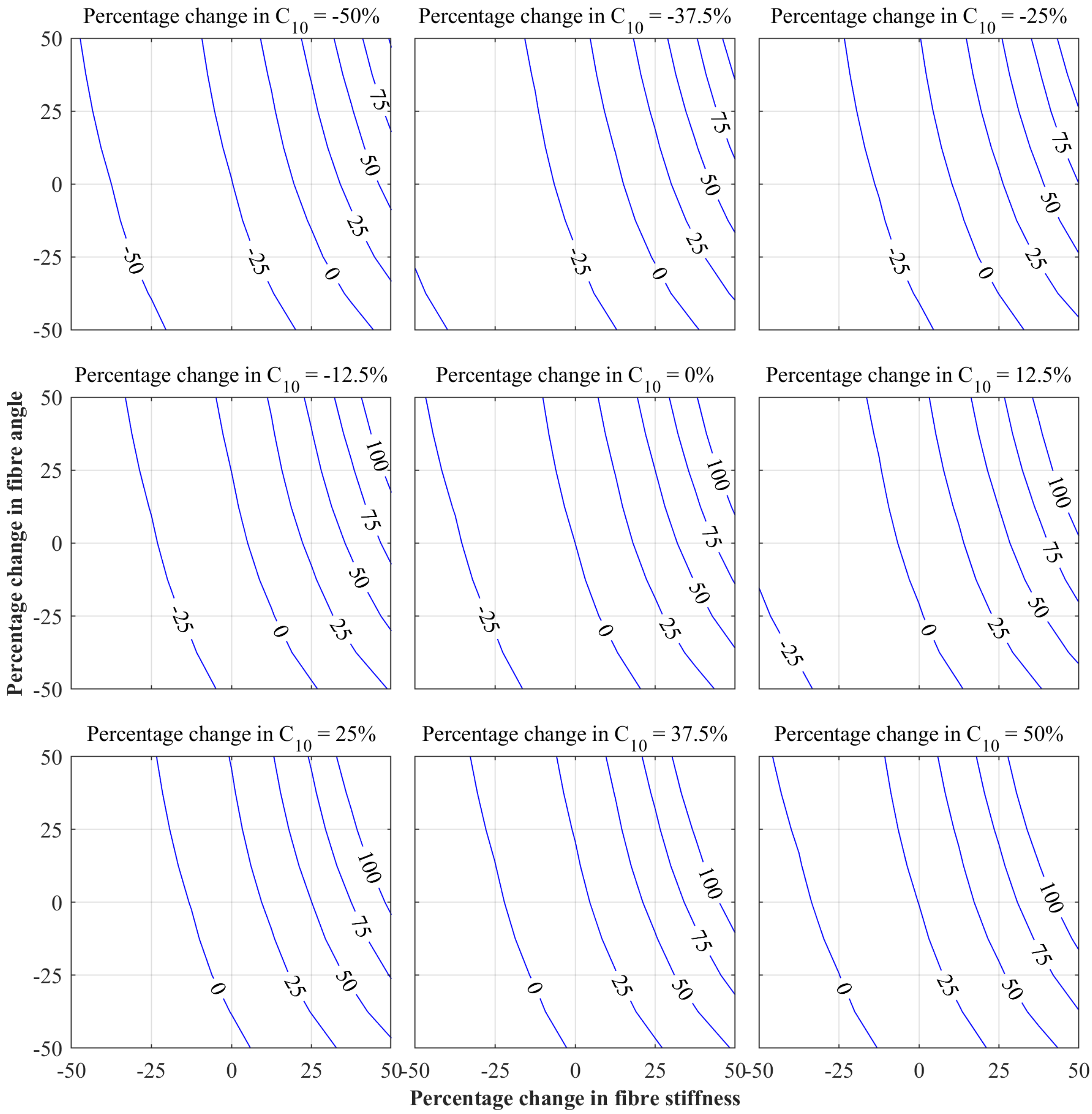

Figure S4. Percentage change in IVD stiffness under lateral bending as a function of changes in the significant parameters.
